# Ulipristal Acetate Efficacy in a Patient with Symptomatic Fibroid and Concomitant Pulmonary Embolism

**DOI:** 10.1155/2020/3249268

**Published:** 2020-02-22

**Authors:** S. von Wunster, P. D'Oria, L. Colonna, G. Patelli

**Affiliations:** ^1^Department of Obstetrics and Gynecology, Alzano Lombardo Hospital, ASST Bergamo Est, Seriate, Italy; ^2^Department of Radiology, ASST Bergamo Est, Seriate, Italy

## Abstract

Ulipristal acetate (UPA) is an effective drug for the treatment of symptomatic uterine fibroids. The drug is highly effective in controlling bleeding control and in the recovery of anemia. Here, we report the case of a woman with severe menorrhagia due to a uterine fibroid and with concomitant pulmonary embolism, a serious life-threatening condition. UPA was shown to be effective in reducing fibroid volume and controlling symptoms, without worsening the underlying embolic disease. No adverse events were observed, and the patient has completely recovered.

## 1. Introduction

Uterine fibroids are common tumors of the uterus occurring in 20–75% of women of reproductive age, and they are rarely symptomatic in about half of the cases [1, 2]. Common referred symptoms are excessive vaginal bleeding and subsequent anemia, which result in a weakening and hampering condition. The selective progesterone receptor modulator (SPRM), ulipristal acetate (UPA), has been introduced in clinical practice as a medical option for treating uterine fibroids: in randomized controlled trials, it has been shown to induce fibroid shrinkage and rapid control of excessive bleeding [3, 4]. The treatment was considered safe. The SPRMs belong to a relatively new molecular class, and in the medical literature, only few data or case reports have been published about their use in patients with fibroids and concomitant morbidities, particularly with concomitant life-threatening medical condition. We report, for the first time, a particular case of a patient with severe menorrhagia due to multiple fibroids, with concurrent pulmonary embolism (PE), safely treated with UPA. The aim is to share safety information when a SPRM is used in patients with a life-threatening concomitant comorbidity, such as PE, due to a thrombotic event.

## 2. Case Presentation

**Figure 1:**
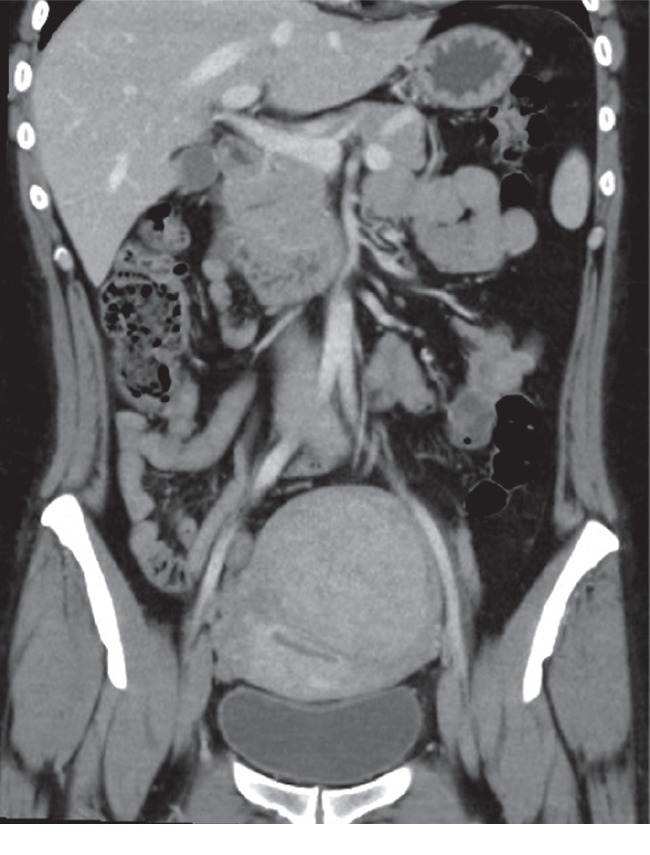
CT scan of abdomen (coronal plane) showing the intramural uterine fibroid.

**Figure 2:**
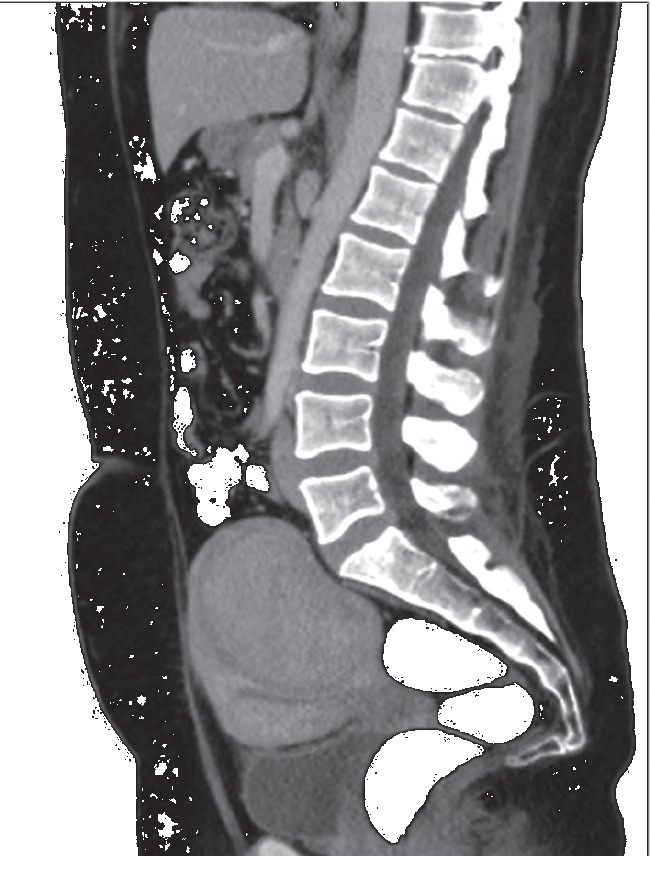
CT scan of abdomen (sagittal plane) showing the intramural uterine fibroid.

**Figure 3:**
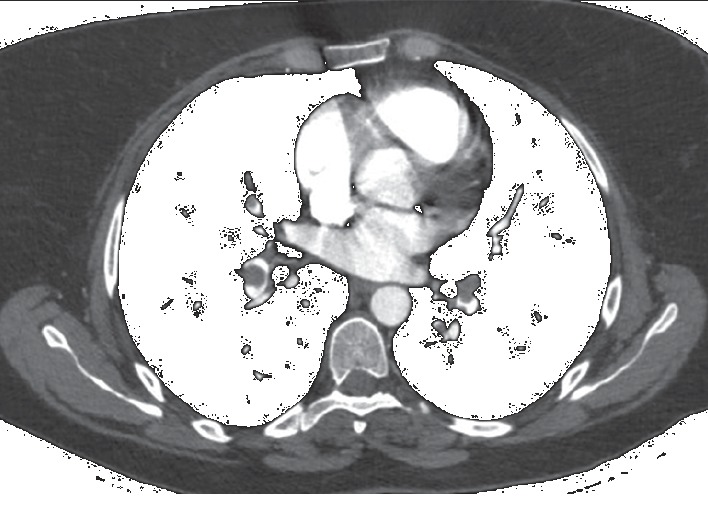
CTPA showing pulmonary embolism.

A 38-year-old woman, overweight (BMI = 28), with a history of anemia and bleeding complaints due to a uterine fibroid (Figures 1 and 2), has been admitted to the Cardiology Department of our hospital after a lipothymic episode. A diagnosis of pulmonary embolism (PE) was performed (Figure 3), due to an embolus starting from a deep vein thrombosis in the right calf, and an anticoagulant therapy with Rivaroxaban was immediately started.

The patient was in a critical condition due to heavy vaginal bleeding and to a very severe anemia (Hb = 6 g/dl) which did not resolve even after transfusion of two blood bags. A symptomatic intramural fibroid (90 × 88 × 89 mm in diameter) was detected and identified as the main cause of symptoms. The proposed emergency hysterectomy was refused by the patient, during the consent informing session, because of the high surgical risks in her critical conditions.

After appropriate counselling, the patient shared the decision to start a daily oral treatment with UPA (Esmya® 5 mg, Gedeon Richter, Budapest, Hungary). Already during the first treatment cycle (12 weeks), an excellent bleeding control was observed; the patient had persistent amenorrhea starting on the fifth day of treatment. Hemoglobin values improved to normal values after three months of treatment with UPA, despite concomitant therapy with high doses of anticoagulants. Concurrently, the fibroid showed a significant reduction in volume. The ultrasonography examination showed a reduction in fibroid diameters, which measured 72 × 70 × 71 mm after treatment. Endometrium was 8 mm depth, with an hyperechoic aspect. The therapy was well tolerated, and there were no adverse events, nor interactions with the concomitant anticoagulant therapy, or exacerbation of underlying pathology.

Given the significant improvement in the patient's clinical condition after the first course of therapy, we decided to continue with a second cycle of daily UPA for three months, starting at the beginning of the second menstrual cycle after the end of the first course of therapy.

At the end of the second course of treatment, normal menstrual bleeding was restored.

**Figure 4:**
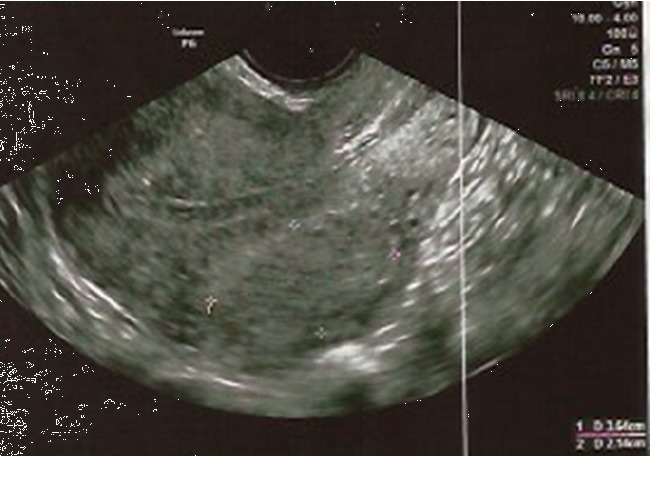
Transvaginal US scan showing the fibroid and the endometrium after two cycles of UPA.

In ultrasound, the fibroid had diameters of 40 × 38 × 39 mm, showing about 90% of volume reduction with respect to baseline. Endometrium has a normal proliferative appearance (Figure 4).

The patient was asymptomatic and in good general condition, and it was decided to follow a “wait and watch” approach. The medical treatment with UPA resulted well toleratant without any adverse reaction and changes in liver enzyme levels.

**Figure 5:**
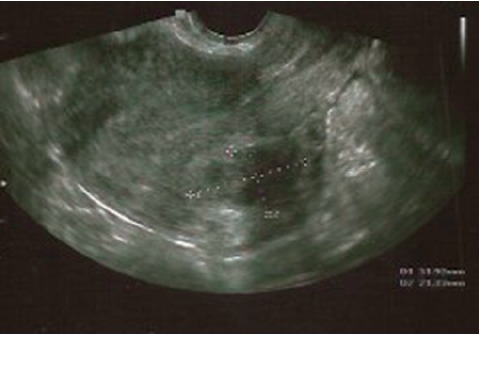
Transvaginal US scan showing the fibroid and the endometrium one year after the end of treatment with UPA.

To date, after more than one year without any treatment for heavy menstrual bleeding, the patient is asymptomatic. At an ultrasound scan performed one year after the end of UPA, the fibroid showed a volume of 31 × 23 × 21 mm and endometrium was normal (Figure 5). No surgery has been performed.

## 3. Discussion

In this case report, it has been reported, for the first time, the effective and safe use of UPA in a women with severe anemia and heavy vaginal bleeding due to a symptomatic fibroid and affected by a concomitant pulmonary embolism, a serious and life-threatening condition.

The patient refused surgical treatment (hysterectomy) due to high surgical risks in relation to both anemia and pulmonary embolism. It is well known that a major surgical intervention, like hysterectomy, in a patient with severe anemia and concomitant pulmonary embolism, is a life-threatening condition that enhances *per se* the mortality risk.

The use of Gonadotropin Releasing Hormone analogues (GnRH analogue) was considered, but these compounds need longer time to induce amenorrhea (2–4 weeks), and furthermore, they may show the well-known flare-up (a huge release of estrogens in a very short time [5, 6]), which could have drastically worsened patient conditions. The patient also resulted to be not eligible for oral contraceptive and estrogen use, a treatment often used for AUB (abnormal uterine bleeding) but contraindicated in case of thrombotic event [7].

During clinical development trials [3, 4], UPA showed a very strong ability to stop bleeding in a very short interval of time (5 days) and also to recover quickly the hemoglobin levels.

The mechanism of UPA is completely different with respect to the GnRH analogues: there is no flare-up, and its action is prevalent as antiprogestogen, antagonizing the proliferative effect of progesterone on fibroid growth [8], with no effect on estrogen receptor activity.

UPA belongs to the SRPMs family, synthetic compounds exerting either an agonistic or antagonistic effect on target tissues, by their binding to progesterone receptors. As selective modulators, they depend on recruitment of cofactors that regulate both transcription along the genomic pathways and nongenomic interactions with other signaling pathways [9]; their effects as agonist or antagonist depend on the coactivator or corepressed recruiter. Despite a number of recent hypotheses, it is not exactly known how SPRMs alleviate menstrual bleeding; it has been suggested that the mechanism underlying the rapid induction of amenorrhea may involve the endometrial vasculature, in particular, the perivascular cells that express the progesterone receptor [10]. SPRMs might induce well-known and typical changes of the endometrium named PAEC (progesterone receptor modulator-associated endometrial changes); these changes are nonmalignant cystic and stromal modifications, which are reversible and benign. These changes return to normal in a few months after the end of therapy [9]. No malignant modification of the endometrium was ever reported during treatment with UPA.

During clinical development of the UPA, no changes in coagulation parameters emerged [9], nor indication of an increasing risk for thrombosis. No contraindication in use in this condition has been reported in SmPC, and for this reason, we decided to consider this treatment [11].

Ulipristal acetate is a very well-tolerant drug with few reported serious side effects during developmental trials. Coagulation parameters were evaluated in detail up to four courses of treatment, and they were found unchanged; no cases of venous thromboembolism were reported in any of the four PEARL studies [9].

In 2018, ESMYA® has been evaluated by EMA's Pharmacovigilance Risk Assessment Committee (PRAC), following reports of serious liver injury. After considering all the evidence, the PRAC concluded that the benefits of the drug outweigh its risks and that the medicine must not be used in women with liver disease. All patients may start treatment only with regular liver tests [12].

In our case, UPA achieved success in stopping bleeding and restoring hemoglobin levels, without interfering with the underlying pathology and anticoagulant therapy. The patient completely recovered without needing further surgery.

## 4. Conclusion

The clinical case presented here highlights the effectiveness of UPA treatment in bleeding control and in reduction of fibroid volume in a woman with symptomatic fibroids and with a serious life-threatening concomitant condition like pulmonary embolism. For the first time, it has reported the use of the drug in this special condition, showing that UPA could be considered a safe option even in women with a pathology in whom traditional therapies are contraindicated or considered at very high risk for life.
